# The role of Allied Health Professions and Nursing Research Internships in developing a research culture: a mixed-methods exploration of stakeholder perspectives

**DOI:** 10.1186/s12961-020-00638-1

**Published:** 2020-10-19

**Authors:** J. Nightingale, S. Fowler-Davis, K. Grafton, S. Kelly, C. Langham, R. Lewis, B. Bianco, D. Harrop

**Affiliations:** 1 Department of Allied Health Professions, Sheffield Hallam University, Sheffield, UK; 2 University of Lincoln, Lincoln, UK; 3 University of Sheffield, Sheffield, UK; 4 Manchester University NHS Foundation Trust, Manchester, UK

**Keywords:** Research capacity development, Internship, research, mixed methods, evaluation, allied health

## Abstract

**Background:**

Developing research capability and capacity within the healthcare professions is a challenge throughout diverse international settings. Within England, the National Institute for Health Research aimed to address these challenges through the Integrated Clinical Academic (ICA) research careers escalator for nurses, midwives and allied health professionals. Poor academic progression has been identified in the advanced stages of the pathway, though progression from the earlier entry point (Internship) has not previously been investigated. A national evaluation of four completed Internship cohorts was undertaken to explore stakeholder perspectives and progression beyond the Internship programme.

**Methods:**

A mixed methods project used sequential qualitative and quantitative data collection phases commencing with two stakeholder focus groups (*n* = 10); the findings informed the development of an online survey distributed to previous cohorts of interns (*n* = 104), their managers (*n* = 12) and academic mentors (*n* = 36). Eight semi-structured interviews subsequently explored the challenges and opportunities afforded by the internships. Thematic analysis was used to review qualitative data from focus groups and interviews, with survey data analysed and displayed using descriptive statistics. Synthesis of data from each phase is displayed within the four level evaluation framework outlined within the New World Kirkpatrick® Training Evaluation Model.

**Results:**

Important regional differences exist yet the internships are highly valued by all stakeholders. Representation varied between different professions, with nursing and some service-based professions poorly represented. All interns successfully completed the programme (*n* = 104), with evidence of positive impacts on interns, colleagues and patient care. Balancing research commitments with clinical activity was challenging; middle managers were seen as gatekeepers to programme success. Progression to the next stage of the ICA pathway is highly competitive and was achieved by only a quarter of interns; access to mentors outside of the funded programme is vital for a successful transition.

**Conclusions:**

The Internship programme succeeds in providing a range of important early experiences in research, though progression beyond the programme is challenging due, in part, to a widening gap between Internship and the next level of the ICA framework. Vital mentorship support to bridge this gap is threatened by a lack of time and funding; therefore, the pursuit of a clinical-academic career will continue to be elusive for many nurses and allied health professionals. A partnership approach to clinical academic support at institutional level is needed with several international models offering alternative strategies for consideration.

## Background

Nursing, midwifery and the allied health professions (NMAHP) are, in combination, the largest staff group within health services, representing potential new capacity for clinical academic research in international health systems [1]. The term ‘Clinical Academic’ has been adopted in the United Kingdom to describe clinicians (doctors and healthcare professionals) who have a role that combines treating patients with undertaking research; they may have employment contracts which span both healthcare and higher education. The combined role encourages research that is cutting edge and supports innovations in clinical practice, thus driving forwards evidence-based practice and evidence-based medicine within the National Health Service (NHS).

Traditional educational approaches including taught provision, informal mentorship and in-job training programmes may be effective in developing research awareness in early career healthcare staff; however, the implementation of clinical academic roles has required a more formalised approach. To facilitate the adoption of clinical academic roles within the NMAHP workforce, the National Institute for Health Research (NIHR) implemented the Integrated Clinical Academic (ICA) careers programme. Practitioners apply for individual awards that progress research knowledge from pre-masters through doctoral study to senior independent clinical research. The Internship is the entry level programme that offers an insight into clinical academic careers; it is open to registered NMAHP staff [2] without any formal research training and who are employed within the NHS. Recruitment is competitive and must show a clear potential for benefiting patients and the public.

Funded by Health Education England (HEE), 10 Internship programmes are delivered by a range of providers, including Higher Education Institutions, HEE subsidiaries and one NHS Trust; all are overseen by HEE on a regional basis in England. The contrasting approaches to commissioning at a regional level result in contrasting delivery (Table 6 Appendix), including bespoke educational packages to small numbers of students, or structured learning offered to higher numbers across a larger geographical area. Applicant selection criteria onto these programmes also varies regionally.

Many health and care organisations and government departments invest in research capacity development in healthcare [3] and it is a moral and ethical imperative to develop, shape and evaluate this activity [4]. The NIHR has invested substantially in training over the last 10 years, though they recognise both structural and cultural barriers to clinical academic progression [5]. While the benefits to individual development can be demonstrated at a service and patient level, progression beyond Masters level for NMAHPs is disappointing [5, 6]. The role of the Internship level in preparing NMAHPs for an ICA career is unknown. In this context and following the completion of four annual intakes (2014–2017), a mixed methods evaluation was commissioned by HEE to examine the impact of the programme in terms of participant progression and stakeholder perspectives.

To inform the evaluation, a review of the literature was undertaken using search terms related to (1) NMAHPs; (2) post-registration research education; and (3) barriers and facilitators to enhancing research capability and capacity. Searches were undertaken on MEDLINE (EBSCO) and CINAHL, with grey literature identified by consulting with expert stakeholders. All papers yielded from the database searches were assessed for relevance during title and abstract screening and further appraisal of context and study design was undertaken during full-text reading. The literature offered context to the primary investigation and was a lens through which to understand policy and planning issues [7] related to embedding a culture of research.

The literature search identified that a clinical academic is a health professional working clinically and involved in academia to try and find better health outcomes for practice (evidence-based medicine or evidence-based practice); they appear to be highly valued by a range of stakeholders in different international settings. Most importantly, clinical academics are seen as the gatekeepers for the dissemination of information by translating research into clinical practice [8–13]. Opportunities for NMAHPs to engage in clinical academic roles in the United Kingdom are limited [14]; fewer than 0.1% of the NMAHP workforce is engaged in active research [15], compared to 5% of the medical workforce. While postgraduate studies are recognised globally as a tool for legitimately strengthening clinical credibility and confidence [16], the United Kingdom NMAHP workforce lags behind countries such as Poland and South Africa in the percentage with a postgraduate qualification [17–19].

Aiming to address these imbalances the Association of United Kingdom University Hospitals generated a target of 1% of allied health staff in clinical academic roles by 2030 [20], though concerns have been raised regarding organisational readiness to achieve the ambitious target [21]. Organisational readiness is noted by Slade et al. [22] to consist of four parallel requirements for embedding a culture of NMAHP research into organisations, as follows:

1. Allied health research policies, regulation, governance and organisational structures that value evidence-based practice;
2. Research capability, receptivity, advocacy and literacy of healthcare leaders;
3. Organisational factors including dedicated staff research positions, time allocated to research, mentoring, professional education and research infrastructure, and partnerships with universities;
4. Individual attributes of clinicians, including research skills and capabilities, motivation, and participation in research teams.

The ‘ideal’ organisational approach presented above aims to create a clinical academic infrastructure in NMAHP services that may in turn sustain growth in research activity [23]. Other strategies for capability and capacity-building focus on embedding dedicated NMAHP research positions [2, 24] and offering research skills training, research bursaries and mentoring [2, 25]. While NMAHPs are clearly interested in clinical academic careers [6], there is often uncertainty about the best way to navigate a clinical academic career pathway [26]. The NIHR Research Internships offer an initial opportunity for NMAHPs to undertake a small research project through funded backfill and mentorship. Effective research supervision enables a tangible outcome toward the next stage of development – usually a master’s degree or application to the next ICA pathway stage (Pre-Doctoral Clinical Academic Fellowship; PCAF) [27, 28].

Internationally, variations on the NIHR Internship model are noted, with performance managed against a range of measures, including completion rates [29]. The majority of research interns have a very positive experience [17, 30–33]. They also report increased confidence and competence, job satisfaction, increased knowledge and skills for hands-on practice, critical thinking in practice, changing practitioner–patient relationships and enhanced communication skills [9, 16–18, 30, 33–39].

Beyond the Internships, the benefits are wide-ranging and long-lasting and include encouraging others, greater involvement in decision-making, increase in motivation, confidence and assertiveness skills, positive impact on service and patient care, increased confidence in writing and speaking skills, and opportunities to teach [9, 16–18, 30, 33–40]. Research opportunities following the Internship include integration into a research team, conducting their own research and being a Principal investigator, or being involved in the development of research projects [9, 17, 33, 40].

Internationally cited structural and organisational barriers to progression from research Internships include a recognition of low pay for research-related roles compared with the expected responsibility [17, 30, 34, 36]. While there are examples of such roles in the United States leading to financial reward during and post completion of internships and post-graduate study [36], the lack of financial support for internships was seen as a fundamental barrier internationally [17, 18, 33, 36]. Additionally, the lack of time release (for both Interns and mentors) and impingement on the work–life balance was often cited as a major conflict [9, 18, 33, 36, 38]. With these potential opportunities and barriers in mind, the evaluation study aimed to explore stakeholder perceptions of the benefits and challenges of the HEE/NIHR Research Internship model, alongside reviewing intern research career progression within and beyond the Internship. This is the first study exploring the Internship programme from a national perspective and will offer funders, policy-makers, educators and research leaders a valuable insight into the potential impact of the programme as a catalyst for promoting research careers and a research culture. Four annual Internship cohorts provide the intended study population; therefore, the career progression findings will relate to the short-to-medium-term impacts of the Internship programme.

## Methodology

### Methodological design

A mixed methodology project combined qualitative with quantitative data collection in a sequential manner; phase 1 (literature review and focus groups) analysis informed the development of tools for phase 2 (survey and interviews). The New World Kirkpatrick® Model [41], developed from a well-established process for recognising the outcomes of training and development [42], was then used as a deductive framework to integrate findings from each phase of the project. The New World model offers subtle changes over the earlier model to address the complexities of modern learning environments [43] and consists of four levels of evaluation (1 = reaction; 2 = learning; 3 = behaviour; 4 = results/impact). The evaluation focused primarily on Level 4 (desired impact) with indicators of success being (1) participant progression (continuation of clinical academic activity) and (2) stakeholder engagement in the programme delivery and outcomes.

The project was submitted to the institutional Research Ethics Committee in two stages for ethical approval (Ethics ID: ER10500858; ER12442076). Participant information leaflets and consent forms were developed and all responses were anonymised; participants were assured of confidentiality and right to withdraw until completion of data analysis.

### Phase 1 data collection and development of phase 2 tools

#### Stakeholder focus groups

Two focus groups of expert stakeholders (*n* = 10) were invited to highlight key issues and topics for inclusion in the survey and interviews. The stakeholders were selected in consultation with the steering group to promote representation from different geographical regions and different stakeholder groups, and included programme commissioners [4], programme providers [3], academic mentors [1] and graduate interns [2]. The focus groups were facilitated by two researchers and were audio recorded and later transcribed.

The key questions asked of the focus group were the following:

1. What are the similarities and differences between the different regional internship programmes?
2. What does success look like for the different stakeholders?
3. What are the potential barriers and enablers to ‘success’?
4. Can we define the ‘key ingredients’ for success?

A thematic analysis was based on coding and categorisation of the focus group data to identify a ‘topics guide’ for the survey and interview (Table 1). The first two themes identified regional and professional disparities and the second two themes referred to the professional barriers and enablers to accessing the 10 regional Internship programmes.

**Table 1:** Stakeholder focus group themes

Key themes	Sub-themes
1. Programme and regional variations:	• No standardisation in recruitment and outcome metrics
	• Lack of communication channels post internship
2. Internship professional differences and characteristics	• AHPs better represented than nurses, some AHPs rarely represented
	• Information dissemination variable across settings (e.g. poor representation in the community setting)
	• Previous Masters experience as a barrier or facilitator contested and debated
3. Barriers to success	• Influence of research culture of organisation
	• Middle managers are gatekeepers to progression
	• Gap between Internship and pre-doctorate NIHR level widening
	• Lack of joined up approaches (various research initiatives)
4. Enablers of success	• Supervisory relationship is key to success and continuation
	• Should be intern driven
	• Showcase impact on service transformation and culture

*AHP* Allied Health Professions, *NIHR* National Institute of Health Research

#### Survey development

The literature and focus group themes were applied to survey development by an expert group who are familiar with Internship training programmes. The questions were grouped into three sections relevant to interns, their managers and their academic mentors, and included both closed and open-ended question groups. Filter questions at the start of the survey enabled the respondents to complete the questions relevant to their group. The survey was hosted on the Qualtrics™ online survey platform (Qualtrics, Provo, UT) [44] and the Qualtrics software-supported data analysis was via descriptive statistics; thematic analysis was undertaken of any open text comments. The survey was piloted by the steering group (including external representatives) and by the research team (including two graduate interns and an Internship mentor) for content, question flow and usability on computers and mobile phone devices. The Director or Course Leader of each of the HEE-funded Internship programmes in England (*n* = 10) was contacted and requested to provide contact emails of interns, managers and mentors of completed cohorts (Internship alumni). An email, with a link to the online survey, was cascaded to all contact emails on the three collated email lists (interns, managers and mentors; *n* = 520), with two subsequent reminders issued.

#### Interview development

Survey respondents were invited to provide their contact details if willing to participate in the next phase of the project. A sampling strategy ensured that interns, managers and mentors with potentially different experiences were approached from different geographical regions. Ten participants were initially contacted for a one-to-one telephone interview guided by a semi-structured interview schedule that explored their experiences of recruitment and admission, the programme itself, and experiences subsequent to the internship. Interviews were audio recorded and later transcribed in full. Thematic analysis identified key themes and quotations [45].

## Results

### Participant demographics

The link to the survey was sent to 317 Interns, 100 mentors and 103 line managers (Table 2); however, a large number of the intern (university) emails were no longer active, resulting in lower than expected response rates. While data was captured on the range of different regions and programmes, the responses for some categories was small, so only the national combined data is presented. The response rate for graduated interns was 33% (*n* = 104/317) and for mentors it was 36% (*n* = 36/100). While this is lower than anticipated, it is nevertheless greater than published averages for online survey response rates (29%) [46]. There was no opportunity to increase response rates as many of the emails supplied were non-functional. However, the intern responses are considered representative as they equate to over one-quarter of the total population of funded interns.

**Table 2:** Survey respondent demographics

Category	Options	Graduated interns^**a**^	Academic mentors	Line managers
**No. of surveys distributed**		**317**	**100**	**103**
**No. of responses received (percentage response rate)**		**104 (33%)**	**36 (36%)**	**12 (12%)**
Gender	Male	14	9	10
	Female	90	27	11
Age group	18–24	1	Not asked	Not asked
	25–34	37		
	35–44	34		
	45–54	28		
	55–64	4		
Agenda for Change Banding (Career level)	Band 5	5	Not asked	Not asked
	Band 6	39		
	Band 7	34		
	Band 8a	11		
	Band 8b	5		
	Band 8c	10		
Start year of the Internship Programme	2014	10	8	Not asked
	2015	12	11	
	2016	34	15	
	2017	48	18	
	2018	n/a	21	
What is your highest qualification (at entry for the interns)	Diploma	5	0	1
	Pre-registration BSc	53	0	3
	PgCert or PgDip	14	0	1
	Clinical MSc	13	2	3
	Pre-registration MSc	3	0	0
	Research Masters^a^	16	5	2
	PhD	0	28	2
	Other	0	2	0
What area do you work in?	Primary and community care	33	4	3
	Secondary care	56	7	7
	Tertiary care	11	2	1
	University	4	22	1
	Other	0	1	0
How many interns have your supported	1	Not asked	17	7
	2		3	2
	3 or more		16	3

*PGCert* Postgraduate Certificate, *PGDip* Postgraduate Diploma

^a^e.g. Masters in Research (MRes) or Masters in Philosophy (MPhil)

The interns were predominantly female (86.5%), equally spread across age range categories from 25 to 54 years. Most interns held a BSc, although 27.8% (*n* = 29) held a post-registration Masters level award. Most interns now worked in secondary care (United Kingdom Agenda for Change Bands 6 and 7); however, a significant number occupied a Band 8 position (senior leadership or consultant practitioner). The interns had variable research experience prior to commencing the internship. The majority of mentors (75.6%, *n* = 28) held PhDs and were University based.

Semi-structured interviews were undertaken with eight participants from different geographical regions, including two line managers, two academic mentors and four graduate interns.

### Framework for data presentation

Analysis of data from Phase 2 (survey and interviews) is combined and presented within the four-level evaluation framework outlined within the New World Kirkpatrick Model. Each level has been interpreted in the context of the internship programme evaluation as demonstrated in Table 3.

**Table 3:** The Kirkpatrick Training Evaluation Model used as a framework for the Health Education England/National Institute for Health Research Internship evaluation findings

Level	Kirkpatrick model focus	Kirkpatrick model question	Internship evaluation focus	Potential topics
1	Reaction	To what degree participants react favourably to the learning event	Stakeholder experiences of the programme	Recruitment and programme experiences
2	Learning	To what degree participants acquire the intended knowledge, skills and attitudes based on their participation in the learning event	Programme outcomes: impact on the individual intern	Completion rates; changes to intern’s knowledge, skills and attributes (e.g. confidence)
3	Behaviour	To what degree participants apply what they learned during training when they are back on the job	Learning transferred and applied into clinical practice	Impact beyond self to others (e.g. research champion, research culture)
4	Results	To what degree the targeted outcome occurs, as a result of the learning event(s) and their subsequent reinforcement	Research career progression and impact within an organisational context	Progression to Integrated Clinical Academic pathway and roles; other indicators of research career progression

### Level 1 analysis – reaction (experiences of the Internship)

**Fig. 1:**
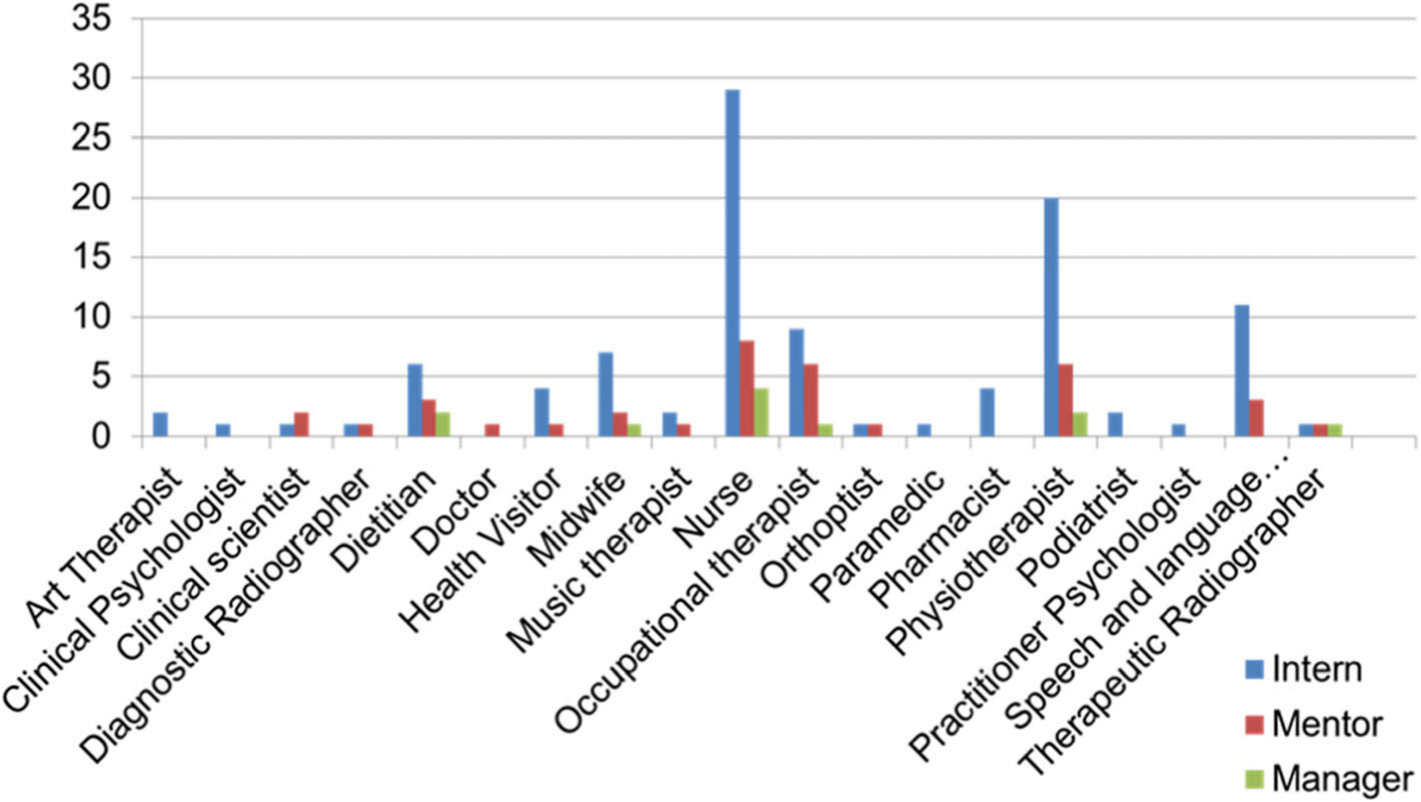
Registered professional groups of survey respondents

The 104 responding Interns included 38.4% registered with the Nursing and Midwifery Council (NMC) and 56.7% registered with the Health and Care Professions Council (HCPC); this latter category included representation from 11 of 13 recognised Allied Health Profession (AHP) groups. The representation of professions across interns, managers and mentors is seen in Fig. 1. Of the 36 mentors who responded, 47% identified as clinical academics and 50% as university academics, with 11% having successfully completed advanced stages of the ICA pathway (Doctoral or Post-Doctoral studies).

All survey and interview participants were very supportive of the concept of the HEE/NIHR Internship. While competition for places was strong, a number of issues regarding fair and equitable access to programmes were raised in both survey and interview responses. Respondents recognised the predominance of nurses and some AHP groups, suggesting that the research-readiness and opportunities afforded to the professional groups differ:

“*… the smaller professions like therapeutic radiographers who don’t have really established networks for research... some of the small professions don’t even know that this* [internship] *exists …*” (Line Manager 1)

Participants highlighted that Internship programmes in different regions had variable admissions criteria, with some considering Masters level study an advantage and others seeing this as a barrier. This was assumed to be because each programme was set up with a different outcome in mind.

“*When I’ve spoken to people outside of the region there isn’t a set transparent clear process on how people get selected for these courses … it could seem quite unfair*.” (Intern 5)

“[Here] *it’s structured slightly differently to in other parts of the country …it was specifically designed to support clinicians to bridge the gap between masters level and PhD level learning…in other parts of the country it’s more of an introduction to research...*” (Intern 8)

Regardless of these perceived inequities, survey respondents were extremely positive regarding the content and delivery of the Internship programmes, though some Interns found the self-directed nature of some programmes challenging. Programmes were largely well-structured with enthusiastic course staff and the outcomes generally met or surpassed their expectations.

The support of the manager and the mentor–intern relationship has the most positive influences on the programme experience, with the main barriers to clinical academic activity as perceived by survey respondents being the lack of time (27.8%, *n* = 29) with some instances of poor line manager support (20.2%, *n* = 21).

“*…other clear enablers are having the time, and I struggled a little bit with that, because I ended up doing most of it outside of my working week…and most of it I did in my own time. And I’m not saying that would stop me doing stuff. It wouldn’t, but at the end it’s a bit of a kick in the teeth … Because even if someone buys your time, they’re actually paying the organisation, and you’ve still got to make that time*.” (Mentor 4)

“*You have to really have that support and buy-in from your line manager…and certainly amongst those* [interns] *they didn’t always have their line manager’s support…it’s almost like it’s Everest before they’ve even got onto the course*.” (Line Manager 6)

Utilising funding for releasing both intern and mentor time (backfill) was consistently cited as a challenge in both the survey and the interviews, although some managers suspected backfill was easier in other professions:

“*I think probably another barrier, and this is quite unique to our profession, is that unlike nursing* [where] *you’ve got quite a big pool of bank nurses that you can just dip into for a shift. It doesn’t really work like that for therapists…*” (Line Manager 6)

“*I think for many clinical managers, and particularly for nursing where there’s such a shortage of nurses in practice, I think sometimes it’s not that the managers don’t want to support these things, it’s they can’t because they just don’t have anybody available* [for backfill].” (Mentor 2)

“*I would say we had underestimated the real pull of the day job here, that we’re here to deliver the clinical service. That even if you’ve got the backfill … it is actually really difficult to practically put into place…*” (Line Manager 6)

Backfill funding varied substantially between regions. Interns on part time contracts were paid additional time to undertake the programme by increasing their hours, though this was not available to full-time employees. However, creative use of funding was discussed by one manager as an alternative to standard backfill:

“*What we did is that the money that we got in we were able to utilise for further study and study days for her other team members, and for courses that we never would have been able to have funded ourselves. So* [the intern]*, she felt great, because she was able to do it, there wasn’t this pressure that she felt she was leaving her colleagues ‘in the lurch’. And her colleagues felt that they were getting something from it as well*.” (Line Manager 6)

Enablers for success on the programme are multi-factorial, with interns and mentors reiterating the importance of the supervisory relationship. Mentors identified challenges of supporting Interns with an unclear research focus, and time and funding limitations. The importance of an internship that is intern rather than mentor driven was expressed by 86% mentors and 52% of interns.

### Level 2 analysis – learning (programme outcomes and impact on the individual)

All responding interns had successfully completed their internship programme (100% completion). Following completion, the graduate interns valued their new research skills and knowledge and the opportunity to engage in research and research networks. The internship had a high impact upon their confidence, patient care and their role, with a lesser perceived impact on their department and their colleagues. Conversely, 92% of managers recognised positive impacts upon the department, the interns’ colleagues and patient care; 75% also recognised positive impacts upon role and skills:

“*I would also say positively that I feel that it’s changed their clinical practice in terms of the fact that although we’re a graduate profession…it just seems to give them that next step, or that next way of thinking*…” (Line Manager 6)

Only a third of the line managers had been involved in project selection; however, 83% identified that the project had been embedded in the department. While there were mixed views regarding the balance of the clinical and academic components, and the impact on self, nevertheless, all recognised the impact of ‘thinking time’ that the internship had afforded:

“*I think it’s an ideal programme and it’s very good for the individual. But I really don’t think there’s much linkage with clinical practice. It’s very academic. The academic is very separate from the clinical practice, and trying to link the two together, it doesn’t really happen. It does seem to run in a very separate environment*.” (Mentor 2)

“*It is a really important time out for people wanting to progress a clinical academic pathway to really have that thinking time to hone their ideas…that was really beneficial.*” (Intern 3)

### Level 3 analysis – behaviour (learning applied into clinical practice)

Expectations of managers was that the Internship would ideally have a positive impact on patient care but also on other colleagues, a so-called ‘ripple effect’.

“*So for me as a service manager I needed to ensure that this was something that would improve patient care ultimately…*” (Line Manager 1)

“*I the line manager hadn’t appreciated the positive… impact that it was going to have on the wider department. And that certainly is something that has now rippled through the rest of the department*” (Line Manager 6)

While NMC-registered interns in the survey had identified greater positive outcomes related to individual roles, skills and confidence, HCPC-registered interns reported having a greater impact on the wider department. Both mentors and interns in the interviews also recognised their impact on their colleagues and saw the intern as a driver for change:

“*I also hoped that it would contribute to improve her overall confidence, and have an impact on the wider team…*” (Mentor 2)

“*I think the internship definitely started to charge organisational attitude to clinical academic opportunities…So taking on the first one and being a role model … I think that’s a privilege…*” (Intern 3)

Stakeholder expectations are not always aligned regarding progression beyond the internship, with some arguing that a longer-term impact on clinical practice could only be achieved if the internship was seen as the start of a research career journey, not the end-point:

“*My expectations as a mentor were that I would be able to take my* [intern] *right the way through their programme… so that they didn’t just stop at the end of the* [internship]. *That was I felt fairly clear that it wasn’t just about this being a one-off internship… It was more about their onward journey as a clinical academic, if that’s what they want to be…*” (Mentor 4)

Conversely, the majority of respondents see the Internship as a valuable early career ‘research taster’ and personal development opportunity; unlike the quotation above, a decision not to continue is acknowledged by some as an acceptable and indeed successful outcome.

“*It’s great to see these internships give people a real chance to understand what early career research looks like, what it is, how it differs from ordinary service development or clinical audits. And it gives them a protected time in which to assess whether or not it is a long-term career for them… And there is no expectation that you continue on that pathway after it, so it’s a taster really that we couldn’t afford to offer without the internship*.” (Line Manager 1)

“*So while it is absolutely being supportive of those people, I think we’ve got that duty as line managers of also identifying those staff that it’s not the right time for them to be doing it.*” (Mentor 6)

“*It helps you to understand really quite well what a clinical academic career might look like. And gives you time and space to think that through and decide whether that’s the right path for you. I didn’t feel any pressure at the end of it to continue in that direction but I felt like I had a lot more ability to make decisions about that*.” (Intern 7)

### Level 4 analysis – results (progression and impact in an organisational context)

**Fig. 2:**
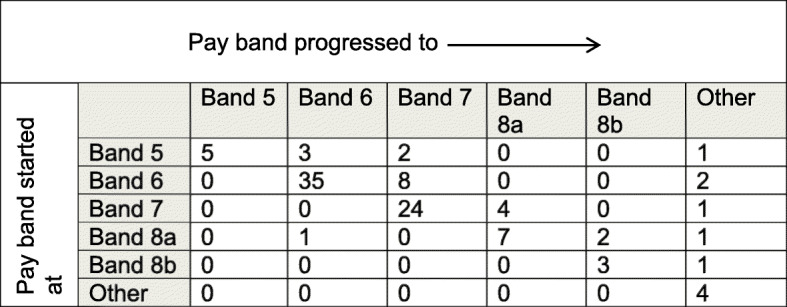
Pay band progression from start of Internship until survey completion

In the United Kingdom, registered nurses, midwives and allied health professionals working clinically within the NHS occupy Agenda for Change pay bandings from Band 5 (early career) up to Band 8 (senior level/consultant practice). The majority of interns occupy Bands 6 and 7; only 19 interns (18.2%) reported progression to a higher pay band since completing the internship (Fig. 2). However, a greater proportion of interns (40%) reported changes in role titles that reflected career progression. There was no specific difference in enhancement of roles between professional groups.

Post-internship, 52% had applied for a higher stage of the HEE/NIHR ICA pathway (Table 4). While the percentage of interns applying from NMC and HCPC backgrounds were similar, more HCPC-registered interns applied for NIHR doctoral level study. Of the 52% of interns who applied, 50% were successful (some were awaiting the outcome of their applications). Interns registered with HCPC had higher success rates than NMC-registered interns (50% compared to 42%).

**Table 4:** Number of interns who had applied for a further programme on the ICA Pathway

ICA Level applied for	All interns completing (***N*** = 100)	NMC (***N*** = 40)	HCPC (***N*** = 59)	Other (***N*** = 5)
ICA MRes	27 (27%)	10	15	2
ICA PCAF	8 (8%)	3	5	0
ICA Doctorate	13 (13%)	3 (8%)	11 (19%)	0
ICA Post-Doctorate	0	0	0	0
Other	4 (4%)	3	1	0
Application to any higher level of NIHR ICA pathway	52 (52%)	19 (48%)	32 (54%)	2
Of those applications, no. of successful awards	26 (50%)	8 (42%)	16 (50%)	2 (100%)

*HCPC* Health and Care Professions Council, *ICA* Integrated Clinical Academic, *NIHR* National Institute for Health Research, *NMC* Nursing and Midwifery Council, *PCAF* Pre-doctoral Clinical Academic Fellowship

Applications to these ICA pathway awards are more likely (and more likely to be successful) from those in the 45+ age band, occupying Agenda for Change Band 7 roles, and who have completed post-registration Masters degrees. The ICA research pathway is not the only funding stream to which graduate interns applied to support the next stages of their clinical academic career; good success rates were evidenced for other funding streams (Table 5).

**Table 5:** Numbers of interns applying for professional body and charitable funding

	Total applying	Total successful	NMC success (***N*** = 11)	HCPC success (***N*** = 14)	Other success (***N*** = 0)
Professional body and charitable research funding^a^	24	18 (71%)	7	11	
Other career pathway funding^b^	21	11 (52%)	3	7	1

^a^Examples: Royal College of Nursing PhD funding, Pharmacy Research United Kingdom Training Bursary, Chartered Society of Physiotherapy Charitable Trust; ^b^Examples: CLAHRCs (Collaborations for Leadership in Applied Health Research and Care), National Institute for Health Research Contingency funding, Health Education England-funded bridging programmes and employer-funded MRes

*HCPC* Health and Care Professions Council, *NMC* Nursing and Midwifery Council

**Fig. 3:**
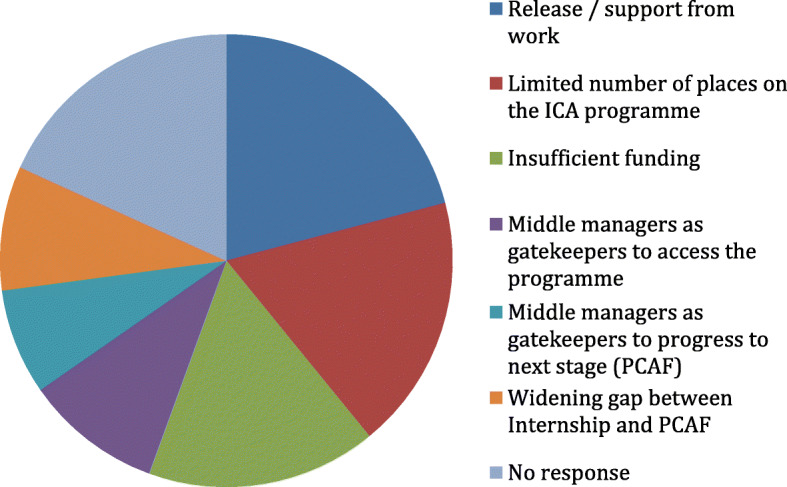
Intern reported barriers to progressing to the next stage of the ICA programme

Overall, 48% of interns had not applied for the next stage of the ICA research pathway. Middle managers were identified by both interns and mentors as gatekeepers; managers were sometimes reluctant to support ICA progression, preferring instead to support further intern applications within their team. Release from work, lack of funding and a shortage of ICA pathway places were also noted by interns as major barriers to progression (Figure 3).

A total of 19% of mentors expressed concerns regarding a widening skills gap between Internship completion and a successful application for the next step on the ICA research career ladder (PCAF). For some interns and mentors this was seen as an insurmountable gulf, which effectively ended their research career progression aspirations. For others, the highly competitive nature of the PCAF application process necessitated further research activity and/or bridging programmes to strengthen their applications. Access to mentors following the Internship was seen as vital for successful progression; while lack of funding may be a barrier to supervision beyond the internship, 56% of mentors said they would support the interns even if they were not paid.

“*And you start this process and you have some good outcomes, and you want to continue* [to PCAF]*. But then you come away slightly disillusioned…Because along the way you’re just given lots of messages of how competitive it is*…” (Intern 4)

“*So my expectations were that it was quite a seamless process. So you could start as being an intern, then progress onto PCAF, and then spend some time and apply for an NIHR grant for a doctorate. So those were my expectations. Having now completed the internship the reality is that the PCAF is a very competitive process, and one would need to complete lots of other activities to build up research experience*.” (Intern 5)

Many Interns commented on the challenges posed by a lack of a defined research career structure and role models that they could aspire to; most do not see how a clinical–academic framework could become a reality in their department or professional group.

“*… they were talking about their own careers as a clinical academic…all of them spoke of the difficult time, and how you have to be so driven….how they felt that due to the framework not being there…… there’s no formal structure in place in how to be a clinical academic*.” (Intern 5)

“*…you kind of get a lot of investment in you and then not much at the end…I can’t find the system to support me*.” (Intern 8)

Despite these challenges, success appears to breed success. Departments who supported previous Interns are more likely to apply for and be successful in future applications. Support for departments new to research is vital for the ICA programme to be embraced:

“*And I think very early on, because we didn’t have the bigger oversight of what the internship leads to in the organisation…it was just quite a low level thing; whereas now we’re promoting internships and pre-doctoral fellowship opportunities and we can now demonstrate to people what the pathway looks like and where our ambition lies*.” (Intern 3)

## Discussion

A mixed-methods approach was employed to evaluate the short- to medium-term impacts of the 10 HEE/NIHR Internship programmes across England. The discussion will be embedded within the four-level framework of the New World Kirkpatrick model [41].

### Stakeholder experiences of the internship

At Level 1 (reaction), intern experiences once enrolled upon the programmes are overwhelmingly positive, as identified in previous studies [17, 30–33]; outcomes generally met or surpassed their expectations. However, the competition for places was strong and many respondents highlighted variability in admissions policies, with Masters’ degrees viewed as either an advantage or a barrier to entry. Regional influences inadvertently created different internship models, yet applicants were unable to ‘cross borders’ to access the model of their choice. While nurses are well represented in this study, the numbers are small in comparison to the size of their profession. Some AHP groups are well represented (Physiotherapy, Speech and Language Therapy and Dietetics), yet others have limited or no representation as indicated by Hiley et al. in their evaluation of the West Midlands clinical academic careers programme [15]. This unrepresentative professional landscape was, according to some stakeholders, exacerbated by ineffective cascading across large organisations, also recognised by Dimova et al. as a barrier to NHS research [47]. Even where organisations spread positive messages about research, the line managers were consistently recognised as the ‘gatekeepers’ to accessing the programme. Service and ward-based professions may have significantly more barriers to engagement in research due to routine operational pressures in addition to national workforce shortages in some professions such as diagnostic radiography [48, 49]. In addition, relatively recent transitions to degree entry present additional challenges for some professions such as operating department practitioners and paramedics. By engaging with the Internship, AHP professions have potential to add value to their services by promoting evidence-based practice initiatives; this increases professional recognition, enables them to exercise greater autonomy, and to profile research within their professional work plans. Conversely, for those groups who remain unrepresented, the transfer and knowledge exchange has not yet been realised; engaging with these under-represented groups will be important in ensuring that the potential impact of the Internship programme reaches across all services, wards and patient pathways.

Within the Level 2 (learning) analysis, 100% of the interns engaging with this study successfully completed their internship programme, unlike some previous studies which report variable completion rates [29]. Learning environments within the different programmes either delivered a research taster, a career escalator through the NIHR research pathway or a combination of the two. This fuelled multiple stakeholder perspectives on the primary purpose of the internship, with success meaning different things to different stakeholders with consequent mixed messages.

Level 3 (behaviour) analysis identified that the mentor-intern relationship has the most positive influences on the programme experience. Barriers to clinical academic activity included lack of time and some instances of poor line manager support. Some Interns were unable to use the opportunity due to ineffective backfill practices; managers also highlighted significant challenges in using backfill funding to release time for full-time employees. In contrast, Interns on part-time contracts had more flexibility; this funding system appears to selectively penalise full time employees who are already at full capacity.

The analysis for Level 4 (results) showcased the outcomes of the programme from the perspectives of all stakeholders. Interns described their learning as gradually becoming ‘research active’. Positive impacts upon the interns (increased confidence, enhanced patient care and more fulfilled roles) were recognised extensively by all stakeholder groups; these impacts are noted in other studies [9, 16–18, 30, 33–39]. Wider impacts include enhanced research culture within their teams, the so-called ‘ripple effect’ identified by other researchers [50], and a more evidence-based approach to professional practices. Given these impacts, it is unsurprising that clinical academics are often seen as the gatekeepers for the dissemination of information by translating research into clinical practice [8–13].

### Progression beyond the internship

Progression through and beyond the internship appears to be dependent on the readiness of the leadership and management in the organisational context to accept and support clinical academic progression as a legitimate and embedded professional activity. Just over one-quarter of intern respondents in this study had successfully applied for the next stage of the NIHR ICA pathway; progression was more likely from those in the 45+ age band, occupying Agenda for Change Band 7 roles, and who had completed post-registration Masters degrees. A report by the Associates of United Kingdom University Hospitals generated a target of 1% of NMAHPS in clinical academic roles by 2030 [20]; while these successful transitions are welcomed, it is concerning that 43% of the Interns surveyed had not applied for the next stage of their ICA development. Cooke et al. had argued that the 1% target was unlikely to be achieved [21] in the absence of sufficient available funded places. Respondents concurred that competition for places was high and viewed as ‘insurmountable’ by some; additional barriers to progression included the absence of research career frameworks, role models and clinical academic recruitment opportunities. Clinical academic posts are indeed rarely advertised [27] and are often held at pay scales that respondents suggest do not reflect the level of responsibility of the role, a trend noted both nationally and internationally [17, 30, 34, 36]. Since completion of the higher level ICA doctoral fellowship programmes, many award recipients had transitioned to an academic position or a clinical post (with no formal sessions for research) [14]. Disappointingly, many returned to the role they held pre-fellowship [14], a worrying indictment for the future of clinical academia.

ICA pathway progression is not the only route to a clinical academic career, with many interns applying for alternative sources of research funding. Career progression was also evident for many interns, achieving a higher pay band and/or enhanced role titles. Clearly evident is the desire and drive shown by many interns to progress their careers yet, for many, the ICA pathway appeared not to be the mechanism to facilitate this. In their evaluation of the West Midlands clinical academic career programme, Hiley et al. described a mentorship gap between the end of intern programmes and submission of publications or next-stage funding applications [15]. This finding was confirmed, with the absence of mentorship and funding to support the transition journey seen as a significant barrier to progression, given the high degree of preparation required for a successful PCAF application. Lack of financial support during and post completion of internships is seen as a fundamental barrier to progression in several other international studies [17, 18, 33, 36]. This transitionary mentorship support, provided currently outside of the funded programmes, relies on goodwill and commitment of senior people who may already have taken on new interns and are therefore at capacity. Mentorship capacity will continue to be a challenge in the foreseeable future as allied health active researchers in practice are low [27], occupying less than 0.1% of the workforce [20].

### Addressing the research skills gap

Significant changes are required at national, professional, institutional and middle management levels to address this research skills gap. Alternative approaches to the HEE/NIHR model provide interesting contrasts; for example, the Victorian Government in Australia have invested in AHP research as an essential component of practice development [51], underpinning evidence-based, person-centred practice. Strategic investment in joint posts within 10 health and care organisations ensures that the ‘clinical academic’ will drive quality and safety improvements in practice. This model contrasts with the NIHR model of individual career development as a mechanism for releasing workforce capacity for research. The Victorian allied health research framework, informed through a systematic review of the literature [22], provides a platform from which research capacity-building strategies can be developed across organisations.

Organisations and managers need to be challenged to commit organisational support to those who are prepared to develop clinical academic careers and this requires investment in the clinical/professional service. Research activity undertaken by clinical academic practitioners needs to be strategically planned in relation to required impacts in quality and service improvement. This ‘payback’ [52], in relation to service outcomes and patient benefits in practice, allows clinical academics to demonstrate a contribution to the population health management at systems level. Within the United Kingdom, the inclusion of research within national Care Quality Commission inspections [53] anecdotally appears to be providing a clearer incentive and narrative for healthcare managers to support their staff to engage in research. This will take time to embed across all institutions and departments; consequently, the pursuit of a clinical–academic career will continue to be elusive for many in the absence of a defined research career structure and visible and proactive role models for all NMAHP.

### Limitations

The survey population included all interns registered on programmes from 2014 to 2018. During this time, a number of changes to the ICA pathway were adopted, in addition to changes to HEE regional boundaries and to programme providers within regions. These changes have all impacted upon the complexity and reliability of data collection and analysis; directly comparing annual cohorts was not feasible.

The invitations to participate in the survey were based on limited and incomplete datasets from 10 different regional programmes; many contact emails supplied to the research team were returned automatically as ‘undeliverable’. While we do not anticipate that this impacted upon the overall survey results, the small numbers in some sub-groups mean that we are unable to draw conclusions about any individual professions or regional programmes. The line manager response was lower than expected and so our data may not be fully representative. While the intern responses were high (*n* = 104), we estimate that this is likely to represent 25% of the total number of Internship registrations nationally, which is not unusual for an online survey applied retrospectively to completed cohorts. It is acknowledged that the interviews did not achieve saturation but were used to offer depth of insight to illuminate many of the survey findings.

## Conclusion

The internship programme is highly valued and has a positive impact upon interns’ confidence, patient care and their workplace. It is evident, however, that many challenges persist in enabling NMAHPs to engage with activities that develop research capacity, even within a well-funded supportive programme such as the HEE/NIHR Internship. In particular, the service provision challenges facing middle management creates barriers to Internship recruitment, effective back-fill and progression beyond the Internship. This facilitation appears to be particularly challenging in small professional groups and in larger service-orientated departments.

The current Internship programme succeeds in providing a range of important early experiences in research, though progression beyond the programme is challenging. A widening gap between Internship and the next level of the ICA framework has been highlighted, with vital mentorship support to bridge this gap threatened by a lack of time and funding. The Internship is the entry-level programme for the ICA career escalator, a model designed to facilitate individual research career development. However, alternative approaches that drive nursing and allied health research development within and between organisations, such as the Victorian model, may support inclusion of a wider range of professions and enhanced progression opportunities. While national Care Quality Commission inspections may provide a clearer research narrative to encourage managers to support their staff to engage in research, the pursuit of a clinical–academic career will continue to be elusive for many NMAHP. Further research is required to explore the longer-term impacts of the Internship on research career progression, including whether clinicians remain in a clinical academic or research related role, and the support they require to do so.

## Data Availability

The survey and interview datasets used and/or analysed during the current study are available from the corresponding author on reasonable request.

## Appendix

**Table 6:** Programme data table: ICA Internship Programmes 2014 to 2018. N.B. Table incomplete as limited responses received from some regions

HEE Region	Midlands & East		South				North		London	
Programme and Region	West Midlands	East of England	Kent Surrey & Sussex	Wessex	South West	Thames Valley	North Pre-2017	North Post 2017	North London	South London
Provider	University Hospitals Birmingham	University of Lincoln	4 Universities: Brighton, Surrey, Kent, Greenwich	HEE Wessex	HEE South West	HEE Thames Valley	HEE R&D NW	Sheffield Hallam University	City University	Kingston/St Georges University
Funding arrangements	£3900 to employer for each intern	£7500 for employer (backfill)	Bespoke spend	Bespoke spend	Bespoke spend	£8000 Bespoke spend	£7500 for employer	£7500 for employer	£5000 backfill; £5000 tuition fees for 2 × 15 credit modules Provider fee – proposal supervision; programme supervision and administration	£5000 backfill; £5000 tuition fees for 2 × 15 credit modules Provider fee supervision; programme supervision and administration
				£10,000 Salary Backfill	£10,000 Salary Backfill		Mentor fee £1000	Mentor fee £1000 Course provision £1500		
	Mentor paid £1000	Mentors paid £1000								
				Supervision £2000 Dissemination cost	Supervision £2000 Dissemination costs					
Total Intern allocated/funded time	30 days over 6 months	48 days total	?	Variable	Variable	?	38 days	38 days	52 days (2 days/wk. for 26 weeks Oct–April)	52 days
Face-to-face independent study contact	Minimum 15 plus 15 days	6 days; 4 action learning sets, 1 celebration day	Varies - monthly if PhD or MRes application	2 days beginning and end and writing for publication			5 days plus 30 days mentored project	4 days plus30 days mentored project	12 days plus 26 day placement	26 days research placement
Academic entry level qualification	Degree or PGDip. Registered healthcare professional as per ICA guidelines	Registered on relevant professional body	Level 6 study within last 5 years	Excludes formal training research May have a pre-reg, PgDip or MSc	Registered 2 years in practice		Not MSc or PhD	Registered on relevant professional body	Degree or PGDip	Degree or PGDip
Accept applicants with: pre-reg Masters	Yes – if minimal research methods input	Yes plus other Masters (if minimal research)					Yes	Yes	Yes	Yes
Accept applicants with post-reg Clinical MSc	Yes	Yes					No	No	No	No
Programme outputs from each intern	Refined research question in preliminary research proposal – potential methods are considered	6000 word (lit review and research report or research proposal) 80% of module mark		Half-way evaluation report	Half-way evaluation report		Academic report 3000–5000 words	Academic report 3000 words	3000–4000 word research proposal including literature review	Preliminary research proposal 2000 words
	Literature review/academic journal	1000 word reflective piece; 20% of module mark	Lit review	Final evaluation report	Final evaluation report		3000–5000 word reflective log	Action plan future development needs	Action plan for CPD derived from reflection on research placement	Group and individual career mentoring to apply for PCAF
Formal accreditation of learning	Yes	Yes	No	No	No	No	No	No	No	No
Success measures	Completion of programme outputs Successful application to PCAF, pre-doc bridging or PhD Promotions, PIs, setting up journal clubs, publications	Passing the module (15 M level credits)	Further study or research funding Project job and work focus change to increase research	Completion of planned studies/pass modules				Complete programme, positive impact upon clinical practice and workplace	Programme completion, grades, development of a high quality research to take forward to MSc/PCAF, etc.	Complete programme, positive impact upon clinical practice and workplace
										Some will apply for PCAF/other research opportunities
								Some will apply for PCAF, etc.		

*CPD* continuing professional development, *HEE* Health Education England, *ICA* Integrated Clinical Academic, *PCAF* Pre-doctoral Clinical Academic, *PI* principal investigator, *PGDip* post-graduate diploma, *R&D* research and development

## Abbreviations

AHP: Allied Health Professions
HCPC: Health and Care Professions Council
HEE: Health Education England
ICA: Integrated Clinical Academic
NHS: National Health Service
NIHR: National Institute for Health Research
NMAHP: nursing, midwifery and allied health professions
NMC: Nursing and Midwifery Council
PCAF: Pre-doctoral Clinical Academic Fellowship
